# A digital therapeutic for management of psychosocial aspects of psoriasis: A pre‐post proof of concept study

**DOI:** 10.1002/ski2.103

**Published:** 2022-04-05

**Authors:** D. G. Fortune, V. Ravnkilde, S. Ryan, B. Ramsay, S. Clough, H. L. Richards

**Affiliations:** ^1^ Department of Psychology University of Limerick Limerick Republic of Ireland; ^2^ Charles Centre for Dermatology University Hospital Limerick Dooradoyle Republic of Ireland; ^3^ Amalgam RX Delaware USA; ^4^ Department of Clinical Health Psychology Mercy University Hospital Cork Republic of Ireland

## Abstract

**Background:**

Despite the psychosocial challenges of living with psoriasis many patients may not be able to access appropriate services to manage these challenges. Mobile health interventions may be helpful as a means to support patients in managing the impact of their condition.

**Objective:**

To conduct a preliminary examination of the feasibility and acceptability of a bespoke psoriasis‐specific digital therapeutic solution (hereafter termed *Allay*), and to provide initial data on psychological changes pre‐post.

**Methods:**

Phase one proof of concept pre‐post study. Eligible patients were provided with *Allay* on their smartphone and assessed at baseline and at 12 weeks on a range of indices of well‐being. Participants experiences on usability were collected by telephone interview at 4 weeks, 8 and 12 weeks.

**Results:**

Out of 66 participants recruited, 59 persisted in using Allay after the familiarisation phase, and 34 participants completed the 12 weeks programme. Participants showed a statistically significant improvement between induction and the end of the 12 weeks programme on Quality of life, Resilience, Perceptions of ‘Overall impact’ of psoriasis, and ‘Emotional impact’. There was a significant change over the course of using Allay for symptoms of depression but not anxiety. While there was an interaction effect of changes in severity of psoriasis symptoms over the course of the study for dermatology‐specific measures, there was no interaction between such changes in psoriasis symptoms and changes in depression, resilience or beliefs in emotional impact.

**Conclusions:**

Study results suggest that the use of Allay as an adjunct to medical management of psoriasis may help patients improve resilience, mood, beliefs about their condition and enhance their quality of life. Given that this is a phase one proof of concept study, and our rates of attrition further research is necessary to examine comparative effectiveness and stability of these findings.

## Graphical abstract

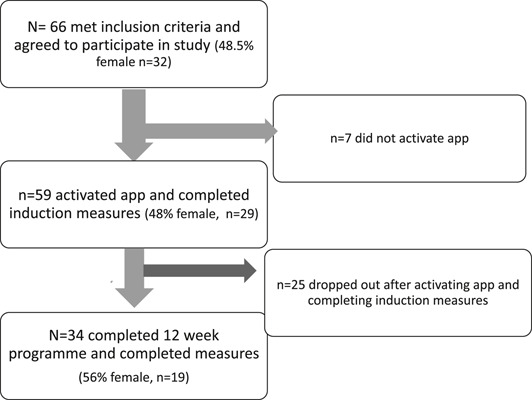

1

**What is already known about this topic?**

- Psoriasis may be accompanied by significant psychological and social morbidity.
- Traditional (face to face) psychodermatology services are limited and underdeveloped.
- Digital therapeutic interventions have been shown to have promise in long term conditions including psoriasis.

**What does this study add?**

- This study examined the initial feasibility and outcome of a digital therapeutic for patients with psoriasis.
- There was a high rate of engagement with the digital therapeutic intervention over the 12 week programme.
- Participants experienced significant positive change in their Quality of life, Resilience, Mood, Perceptions of ‘Overall impact’ of psoriasis, and ‘Emotional impact’, but not in anxiety, or positive mental health.
- Digital therapeutic approaches are deserving of further examination as an adjunct to medical management of psoriasis.

## 2 INTRODUCTION

Psoriasis is currently recognised as a significant health problem,^1^ causing a degree of disability comparable to a number of other major medical diseases.^2^ Patients with psoriasis commonly report feelings of stigma, shame and embarrassment,^3^ which may lead to clinically‐recognised aspects of psychological distress, such as anxiety and depression, and which may also in turn interfere with adherence to treatment^4^ or reduce treatment effectiveness.^5^ It is becoming increasingly recognised that the psychological and social impact of skin diseases requires a significant increase in appropriate psychological support services to meet the needs of patients.^6^, ^7^

These kinds of supports, however, are costly to provide from a health services perspective and the scarcity of such services means that many people who may benefit from such interventions may be unable to access them.^7^, ^8^, ^9^

While it is generally recognised that psychosocial interventions can be helpful for patients, there exists significant diversity in psychological and therapeutic approaches for psoriasis which can make it challenging to perceive which kinds of therapeutic intervention are most effective. For example, Xiao and colleagues^10^ in their systematic review and meta‐analysis suggested that the most effective approaches tend to be cognitive behavioural therapy (CBT) approaches. Qureshi et al.^11^ in their meta‐analysis reported the effectiveness of a number of approaches that could be included within the broader umbrella of CBT and the so named ‘third wave’ therapies including CBT, mindfulness‐based therapies, motivational interviewing, as well as some educational and interdisciplinary interventions. Zill et al.^12^ found positive effects of psychosocial interventions in their review and meta‐analysis and reported that the interventions studies that were of sufficient quality to enter their meta‐analysis primarily used cognitive behavioural techniques, approaches based on mindfulness and meditation or emotional narrative techniques. Thus while there is some diversity in approaches, psychological interventions built around a clinician‐led cognitive‐behavioural model of functioning alongside medical management have been shown to most consistently have merit in people with psoriasis.^12^, ^13^, ^14^, ^15^ In addition there is also recent evidence of the utility of third wave approaches, in particular mindfulness and acceptance‐based or compassion focussed approaches which involve developing a more compassionate style of self‐to‐self relating.^16^, ^17^, ^18^

Hover, a substantial proportion of patients who could benefit may not attend clinics due to the very issues that such adjunctive programmes are designed to target (e.g., embarrassment, stigma).^19^ Development and implementation of non‐traditional methods of enabling access to psychological tools for patients with psoriasis has included the use of internet delivered CBT which has shown some promising results.^20^, ^21^ This research has also indicated that while tools and techniques can be readily integrated into such on‐line platforms for self‐help, the importance of a relationship with the ‘internet therapist’ remains key.^13^

The development of digital health technologies represents a substantial improvement in accessibility options for patients. Digital health interventions have been defined as products or services that use computer technology including mobile devices and web‐based applications and encompasses electronic health (eHealth) and mobile health (mHealth) interventions.^22^ Digital health includes the use of wearable devices, mobile health, telehealth, health information technology, and telemedicine. Mobile digital devices such as the smartphone provide a highly convenient and immediately accessible platform through which some psychological tools may be made available to patients. One recent study in patients with psoriasis found that participants who were provided with education and support through a disease monitoring smartphone application significantly reduced both anxiety and depression over the course of 60 weeks.^23^ The need to examine the acceptability and clinical utility of providing such adjunctive, always available psychological support for patients through a digital therapeutic solution is apparent. This is highlighted in particular given the growing body of literature throughout the chronic disease domain showing in many cases, similar outcomes for digital health when compared to face‐to‐face interventions.^24^, ^25^

The current study set out to provide an initial examination of a comprehensive psychologically‐informed smartphone application developed for patients with psoriasis, hereafter named *Allay.*

*Allay* is a smartphone application which was developed using a patient‐centred, iterative process to create, develop, refine and implement it. This was a tripartite process where patients, clinical researchers in psoriasis and psychology, and experienced mHealth app developers provided their unique knowledge and perspectives into the creation of *Allay*. This process was undertaken to ensure that the content of the digital therapeutic app was likely to be acceptable to patients with psoriasis generally, that it was firmly evidence‐based, and reflected best clinical practice in management of patients with psoriasis. Allay is based upon cognitive behavioural and third wave therapeutic principles including cognitive behavioural tools and techniques, mindfulness, compassion and gratitude, and Inquiry‐Based Stress Reduction (IBSR).

Our principal aims were to: (1). To examine whether the smartphone application *Allay* could improve patients' self‐reported quality of life, mental health, resilience, and beliefs about psoriasis, and (2). To report information on the acceptability and usability of *Allay* in this patient group.

## 3 METHODS

### 3.1 Study design

The study is a phase one proof of concept investigation of a smartphone digital therapeutic intervention for patients with psoriasis. The study examines intention to treat data and data on intervention completers.

### 3.2 Participants

Patients who met inclusion criteria were recruited by research staff from patients attending the Dermatology centre at University Hospital Limerick, Ireland (*n* = 55) and at Forest Hills Dermatology, New York (*n* = 11). Patients were excluded if they: (a) had guttate, erythrodermic or generalised pustular psoriasis to reduce as far as possible potential confounding effects of disease heterogeneity, (b) were unable to use the app due to literacy, cognitive impairment or physical challenges; thus patients who reported problems with reading and writing that would affects their use of smartphones were excluded from the study, (c) were planning on starting or stopping a new biologic agent for the treatment of Psoriasis during the 12 weeks intervention period, (d) were engaged in active or ongoing CBT‐like therapy (digital or in person) at screening, (e) had current or recent (6 months) substance misuse, (f) had significantly worsening psoriasis at induction to the study, (g) were currently experiencing or had a history of suicidal ideation as assessed at screening.

Participants routinely attending dermatology services for treatment of their psoriasis and who met the study inclusion criteria were identified through research staff attached to the study and provided with information in relation to the study. Participants who expressed an interest were then spoken to by the research staff attached to the project, and further information provided as requested. Participants provided written informed consent.

The current paper provides data on all participants inducted, and on the 34 participants who completed the 12 weeks programme (intervention completers). The study was approved by University of Limerick Hospitals research ethics committee (ref no: 011/018) and all participants provided written informed consent.

**FIGURE 1:**
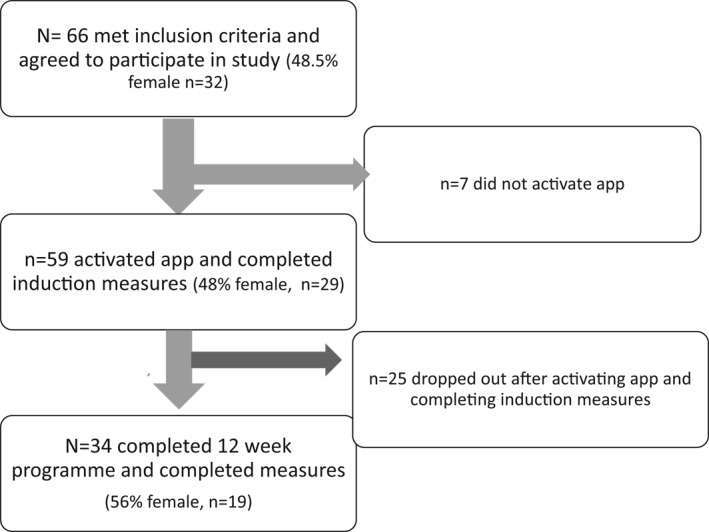
Flow of participants to the study

At induction to the study, participants from both centres were broadly similar in age (US mean 40.0 years SD = 12.58, Republic of Ireland mean 38.9 years SD = 16.22). Participants from the New York centre reported weaker illness coherence beliefs (*t* = −2.99, *p* = 0.01). There were no other differences between patients from the two centres (*t*'s < 1.59, ps > 0.12). Figure 1 below details the flow of participants to the study.

### 3.3 Outcomes assessed

#### 3.3.1 Quality of life

The Dermatology Life Quality Index (DLQI) ‐ a validated 10 item dermatology‐specific questionnaire was used to assess Quality of life.^26^ Cronbach's alpha was 0.94 in the current study.

#### 3.3.2 Resilience

Resilience was assessed through use of the 6 item Brief Resilience Scale.^27^ Internal consistency was acceptable in the present study (Cronbach's alpha = 0.81).

#### 3.3.3 Patients personal beliefs about psoriasis

Patients beliefs about their psoriasis was assessed by the Brief Illness Perception Questionnaire (B‐IPQ).^28^ The Brief IPQ (B‐IPQ) assesses Perceived Consequences of psoriasis, Timeline (acute‐chronic), Perceptions of Personal Control, Treatment Control, Identity (symptoms), Concern about the illness, Coherence of the illness and Emotional representation. This measure uses one single item on a scale from 0 to 10 to assess each dimension of belief/perception, where higher scores on these individual beliefs/perceptions indicate stronger perceptions held by patients along that dimension. Given the individual items assess distinct perceptions about psoriasis, the B‐IPQ is not summed to yield a total scale score.

#### 3.3.4 Severity of psoriasis symptoms

Severity of psoriasis symptoms were assessed by the Psoriasis Symptom Inventory (PSI); an 8‐item patient‐completed measure of common symptoms of psoriasis.^29^ Cronbach's alpha = 0.93.

#### 3.3.5 Mental health

The Patient Health Questionnaire (PHQ) 4^30^ was utilised to measure patient's mental health. This is a 4 item measure comprising 2 diagnostic core items (DSM) for depressive disorders, and 2 core criteria for generalised anxiety disorder. In the current study, Cronbach's alpha for the PHQ‐4 was 0.83.

#### 3.3.6 Mental health continuum‐ short form (MHC‐SF)

The MHC‐SF^31^ is a 14 item measure of positive mental health reflected in the subscales of emotional well‐being, social well‐being, and psychological well‐being. Cronbach's alpha was 0.93.

### 3.4 Description of allay

**FIGURE 2:**
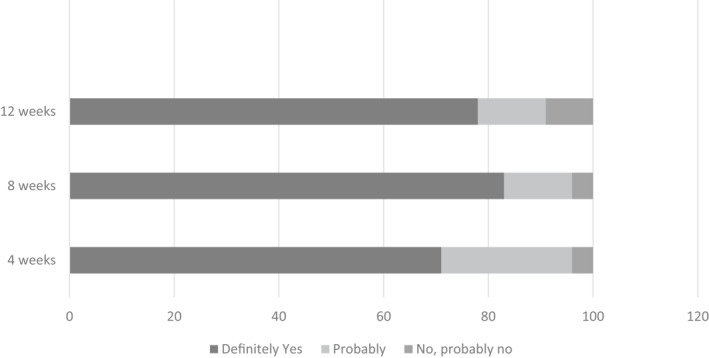
Request to use Allay on completion of study (treatment completers)

*Allay* is a 12 weeks bespoke digital therapeutic intervention for patients with psoriasis and is designed as an adjunct to medical management of the condition (Table 1). Participants were provided with Allay on their smartphone or similar device and continued under the routine care of their dermatology service as normal. The CBT tools were developed from our previous face to face work with patients with psoriasis.^13^, ^14^ IBSR^32^ and positive psychological tools including gratitude and forgiveness could be chosen by participants as tools to manage the impact of their psoriasis. Figure 2 below shows an example of some of the content of Allay.

**TABLE 1:** Content of the Allay m‐health intervention

Description of intervention	Allay is a 12 weeks bespoke digital therapeutic intervention for patients with psoriasis and is designed as an adjunct to medical management of the condition.
Activities	(1). Awareness activities, that promote awareness of the interacting roles of thoughts, emotion, behaviour and the self in psoriasis.
	(2). Gratitude activities, that build towards a conceptualisation of best possible selves.
	(3). Forgiveness activities centred on considering and engaging with aspects of compassionate thoughts and acts.
	(4). Inquiry‐based stress reduction, which through identifying and using the meditative practice of self‐other perspectives supports participants to systematically identify, question and manage unhelpful self‐referent thoughts and beliefs.
Support	A ‘therapist object persona’ was integrated into Allay to help participants with the completion of modules, and to support their engagement with the app more generally. A chat‐bot experience was integrated for the majority of CBT and positive psychology activities to promote ease‐of‐use and help with motivation.
Patient‐centeredness	Participants could also skip or bypass particular features within Allay if desired to meet their individual needs.

Abbreviation: CBT, cognitive behavioural therapy.

Similarly to CBT, IBSR shares the idea that dysfunctional cognitions are a principal factor in the emergence of distress but instead of the use of evidential argument and reasoning, IBSR addresses cognitive reframing through skills and principles of mindfulness and cognitive‐affective self‐inquiry. Thus Allay utilises a holistic and patient centred approach utilising standard CBT, and interventional tools including gratitude, forgiveness and mindfulness from evidence‐based third wave psychological therapy approaches.

#### 3.4.1 Acceptability and engagement

Examination of participant's experiences of usability and acceptability were conducted by telephone interview at 4 weeks, 8 weeks and at 12 weeks. Questions were related to: (a) any challenges in using the app, (b) any changes that the participant would like to see to make it better for them/more user‐friendly, and (c) what they found most helpful about it.

Participants were also asked whether they intended to continue to use Allay after the end of the study. Acceptability and engagement were also assessed by the use of persistence data, assessing the frequency of use of Allay over the 12 weeks of the intervention.

#### 3.4.2 Statistical analysis

QoL (DLQI) scores and Mental Health (PHQ‐4) scores showed positive skew (Shapiro Wilks test DLQI = 0.86 *p* = 0.01; PHQ 4 = 0.90, *p* = 0.01). Due to the non‐normal distribution of these variables, they were transformed by computing a log10 function to render them acceptable for parametric analysis. These two transformed variables were used in all further inferential analyses.

Analysis of all patients inducted (first observation carried forward) and an analysis on intervention completers were used. To control for multiple tests, the more restrictive significance criterion was set at *p* = 0.01. 95% confidence intervals (CI) are provided as appropriate. Repeated measures analysis of covariance  was used to examine the effects of change scores in symptoms of psoriasis on outcomes in participants who completed their 12 weeks programme. Effect sizes are judged small when between 0.2 and 0.5, medium when between 0.5 and 0.8, and large when >0.8.^33^

## 4 RESULTS

### 4.1 Sample characteristics

Of the 66 patients inducted, 50% (*n* = 33) were men, 48.5% (*n* = 32) were women, and one participant did not state gender. Participants were aged between 18 and 64 (mean 39.1, SD 15.27).

At induction there were no significant differences on baseline measures between patients who went on to complete their 12 weeks programme, and patients who were inducted to the programme and did not complete (ts < 1.51, *p* > 0.13). Proportionally, more women than men completed the study (*n* = 19 vs. *n* = 15) however the difference was not statistically significant (*χ* ^2^ = 0.97, *p* = 0.32).

At induction the only gender difference was that women reported a stronger understanding of their psoriasis than men (*t* = −2.66, *p* = 0.01; 95% CI −2.65 –0.374).

### 4.2 Effectiveness data

#### 4.2.1 All patients inducted

Patients showed significant changes from baseline to end of intervention in the extent to which they perceived that psoriasis affected them emotionally (*t* = 2.94, *p* = 0.005; 95% CI 0.27 to 1.46; *d* = 0.62), and in the impact of their psoriasis (*t* = 3.93, *p* = 0.001; 95% CI 0.47 to 1.44; *d* = 0.73; Table 2), in levels of resilience (*t* = −2.52; *p* = 0.01; 95% CI −0.29 to −0.03; *d* = 0.45), symptoms of depression (*t* = 2.92, *p* = 0.005; 95% CI 0.005 to 0.11; *d* = 0.66) symptoms of psoriasis (*t* = 2.58, *p* = 0.01; 95% CI 0.38 to 3.05, *d* = 45), and QoL (*t* = 3.10, *p* = 0.003; 95% CI 0.04 to 0.21; *d* = 1.80; Table 3).

**TABLE 2:** All patients inducted pre‐post mean (SD) and tests of significance for beliefs about psoriasis (B‐IPQ)

Measure	Induction	End of intervention (12 weeks)	95% CI	t	*p*
B‐IPQ life impact	6.10 (2.80)	5.10 (3.20)	0.47–1.46	3.93	0.001
B‐IPQ time line/Chronicity	8.22 (2.47)	8.61 (2.38)	−0.75 to −0.05	−2.29	0.025
B‐IPQ personal control	4.85 (2.87)	4.89 (2.82)	−0.45 to 0.38	−0.15	0.87
B‐IPQ treatment control	8.02 (2.04)	8.18 (2.02)	0.17 to −0.51	−0.91	0.36
B‐IPQ symptom identity	5.71 (2.87)	5.50 (3.03)	−0.47–0.89	0.61	0.54
B‐IPQ concern	5.85 (2.89)	5.27 (3.08)	−0.32–1.02	1.05	0.29
B‐IPQ coherence	7.37 (2.33)	7.73 (2.07)	−0.77 to 0.07	−1.68	0.10
B‐IPQ emotional representations	6.18 (3.02)	5.13 (3.17)	0.27–1.46	2.94	0.005

Abbreviations: B‐IPQ, Brief Illness Perception Questionnaire; CI, confidence intervals.

**TABLE 3:** All patients inducted pre‐post mean (SD) and tests of significance for Quality of life, resilience, mental health and well‐being

Measure	Induction	End of intervention (12 weeks)	95% CI	t	*p*
Dermatology life quality (DLQI)	0.78 (0.40)	0.65 (0.47)	0.04–0.21	3.10	0.003
Resilience (BRS)	3.06 (0.69)	3.23 (0.66)	−0.29 to −0.03	−2.52	0.01
Mental health (MHC‐SF)	48.19 (14.01)	49.50 (14.46)	−3.62–1.01	−1.13	0.26
Anxiety (PHQ‐4)	0.39 (0.27)	0.37 (0.27)	−0.04–0.07	0.42	0.67
Depression (PHQ‐4)	0.29 (0.15)	0.21 (0.13)	0.02–0.11	2.92	0.005

Abbreviations: BRS, Brief Resilience Scale; CI, confidence intervals; DLQI, Dermatology LIfe Quality Inde; MHC‐SF, Mental Health Continuum‐Short Form; PHQ‐4, Patient Health Questionnaire ‐ 4.

#### 4.2.2 Intervention completers

Results for treatment completers also showed a significant improvement and medium to large effect size in their psoriasis‐specific quality of life (*t* = 3.31, *p* = 0.002**;** 95% CI = 0.09–0.40; *d* = 1.06), and a medium effect size for changes in Resilience (*t* = −2.62; *p* = 0.01; 95% CI = −0.55 to –0.07; *d* = 0.66). As before, changes in positive mental health (Mental Health Continuum‐Short Form) was not significant (*t* = 1.23, *p* = 0.23).

In terms of beliefs about psoriasis, significant changes pre‐post was observed for emotional perceptions (*t* = 3.13, *p* = 0.004; 95% CI = 0.57–2.70; *d* = 0.96), and changes in the strength of their belief that their life was adversely affected by their psoriasis, showing a large effect size (*t* = 4.42, *p* = 0.001; 95% CI = 0.98–2.65; *d* = 1.18).

While symptoms of anxiety as assessed by PHQ‐4 did not show evidence of significant pre‐post change in scores (*t* = 0.42, *p* = 0.67), there were clear reductions in symptoms of depression over the course of Allay from baseline to the end of the intervention (*t* = 3.09, *p* = 0.004; 95% CI = 0.04–0.21; *d* = 0.96). Furthermore, using recommended cut‐offs on the PHQ‐4 for screening cases of depressive disorder, at baseline 23.5% of the sample scored above the screening cut‐point for depressive disorder. At the end of the intervention, this had significantly reduced to only 2.9% of cases scoring above the cut‐point on depression.

As would be expected, patients also reported a significant reduction in the symptoms of psoriasis from baseline to end of intervention (*t* = 2.70, *p* = 0.01; 95% CI = 0.80–5.75; *d* = 0.94).

When symptom changes in psoriasis was entered as a covariate in the model, there was an interaction effect for stronger beliefs about the impact of psoriasis on life (F[1,31] = 12.20, *p* = 0.002; *d* = 1.28), and Dermatology‐specific QoL (F[1,31] = 60.92, *p* = 0.001; *d* = 2.84), both demonstrating large effect sizes. By contrast, there was no interaction effect of reduction in psoriasis symptoms on reduction in symptoms of depression (F[1,31] = 0.03, *p* = 0.86), on changes in the strength of beliefs in the severity of an emotional impact of psoriasis (F[1,31] = 0.85, *p* = 0.36), or on resilience (F[1,31] = 3.33, *p* = 0.08).

### 4.3 Acceptability, engagement and usage

**FIGURE 3:**
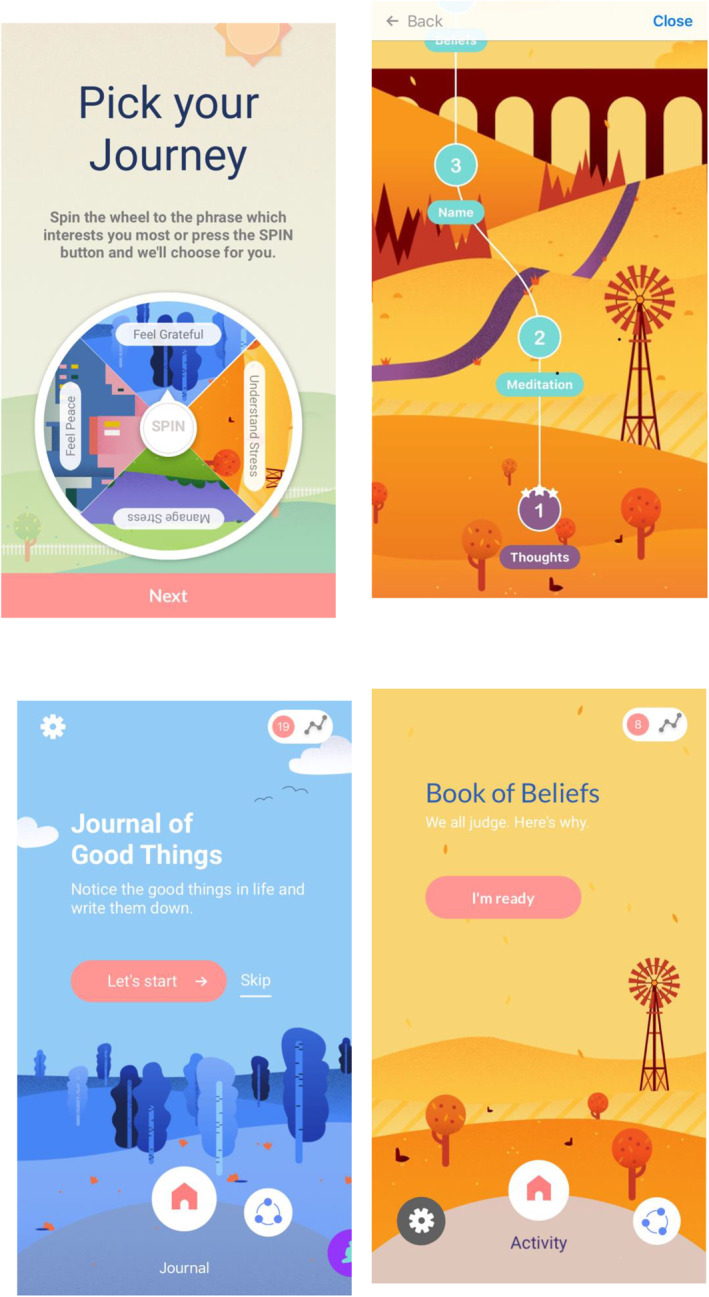
Visual examples from Allay

At 4 weeks into their programme, 70% of intervention completers requested to continue their use of Allay after the end of the study, at 8 weeks 82.6% requested to continue, and at 12 weeks 78% of participants requested continued use of Allay after their programme had ended (Figure 3).

Eighty one percent of participants who began Allay persisted with the intervention through the first 4 weeks. The most common pattern of usage of the app was twice per day (range 0.7–3 times per day). Across the 12 weeks of the study, women tended to use Allay more frequently than men, ranging from 3.1 times more frequently in weeks 1–4, 1.9 times more frequently in weeks 4–8, and 4.2 times more frequently in weeks 8–12. In the telephone check‐ins, the psychological tools that were most consistently reported as helpful by participants across the 12 weeks of study participation were Awareness (CBT; *n* = 29; 85.3% of intervention completers consistently reported this as very helpful), Inquiry‐Based Stress Reduction (IBSR; reported by *n* = 25; 73.5% of intervention completers as very helpful) and Gratitude activities (reported by *n* = 20; 58.8% of intervention completers as very helpful). In terms of technical usability, participants reported accessibility, ease of navigation and clarity (*n* = 27; 79.4%) and flexibility of choice as to the order of tools (*n* = 24; 70.5%) to be most helpful over their 12 weeks experience with Allay.

## 5 DISCUSSION

This is one of the first studies to examine the effect of a bespoke evidence‐based mHealth‐deployed psychological intervention specifically developed for patients with psoriasis. Results of this study suggest that whether an intention to treat analysis or an analysis on intervention completers was utilised, results were substantially similar with patients reporting improvements in quality of life, in emotional perceptions, resilience, and in symptoms of depression.

Interestingly even in cases where patients experienced significant reduction in the severity of symptoms of their psoriasis, there was no observed interaction between changes in depression, resilience or beliefs about the emotional impact of psoriasis. By contrast, there was an interaction effect of changes in psoriasis symptom severity on dermatology‐specific QoL and beliefs about the impact of psoriasis on the person's life. This may suggest an additional level of specificity and validity of the *Allay* intervention: namely that interactions with changes in severity of psoriasis were in the areas of dermatology‐specific assessments related to QoL, while the absence of interaction with changes in psoriasis severity was in relation to more general psychological factors (depression, resilience and beliefs in emotional impact). Interestingly we have previously found a divergence between changes in psoriasis‐specific and more generic aspects of psychological functioning following successful pharmacological treatment of psoriasis.^34^ This particular result of the current digital therapeutic intervention add to the data on this interesting effect.

The observed changes in positive aspects of mental health and other aspects of the personal model held by patients such as beliefs about treatment effectiveness did not reach statistical significance. Beliefs such as these have previously been shown to be very challenging to modify even in individual or group psychological therapy. For example, a previous face to face trial of a CBT intervention for psoriasis targeting such beliefs^14^ suggested that lack of change in beliefs about disease course or chronicity may be due to the observation that such beliefs are grounded in patients' experiences of the vagaries of their condition and thus may be based on appropriate evidence which is unlikely to change as a result of intervention.

Interestingly an internet based study reported a significant effect of their internet delivered CBT programme on anxiety and QoL but not on depression,^20^ whereas we found an effect on depression and Qol but not on anxiety. While different measures of anxiety and depression were used in both studies, nonetheless the reasons for this difference in effect are unclear. We expected a similar positive impact of the intervention on anxiety, however perhaps the content of Allay favoured a focus on mood rather than anxiety in the current sample. Additionally perhaps anxiety, particularly anxiety that has a significant social evaluative component experienced by patients with psoriasis, may require additional methods to support the patient to more mindfully disengage from attentional investment in social situations^35^ where continued engagement‐avoidance may serve to reinforce unhelpful anxiety driven perceptions and beliefs.

### 5.1 Limitations

This study has some limitations. Firstly, this study was conducted in an outpatient hospital setting and therefore may not generalise to the population of patients with psoriasis who may be treated at a primary care level. Secondly this was a pre‐post study without a randomised control group, and we cannot state with certainty that the observed changes were solely due to participation in Allay: a randomised controlled trial is now required to provide definitive data in this respect. Thirdly, our rate of attrition, while not unusual for this type of study, was higher than we would have liked. Attrition tends to be quite high from mHealth interventions generally; meta analytic studies show attrition averaging 43%^36^ and drop out in similar face to face psychological therapies in Psoriasis approach 40%. We also excluded patients with obvious health literacy concerns as appropriate for this stage of study, however future work with larger sample sizes may relax this sampling requirement. Finally we do not have longer term follow‐up data that may have permitted examination of the continuation of the observed improvements in participants' outcomes. Further research is required to examine the longevity of these effects.

### 5.2 Conclusions and implications for research and practice

The increasing need to develop and test patient‐centred psychological interventions in dermatology has been the focus of priority setting partnerships for the health service.^37^ While traditional ‘face to face’ psychodermatology services are both limited and underdeveloped, there would appear to be some scope for use of psychological interventions that are deployed through m‐Health frameworks. However, in many studies these ‘psychological interventions’ may solely include exercise interventions, educational interventions, and many other types of intervention delivered in an m‐Health format within the catch‐all category of ‘psychologically informed’ interventions. This can be confusing for patients, clinicians, and researchers. In the current study we used evidence‐based psychological tools developed using a tripartite process with patients, clinical researchers in psychology and psoriasis, and experienced mHealth app developers. In this initial phase one study *Allay* has been shown to potentially hold some promise as an adjunct to treatment as usual for patients with psoriasis.

There is a clear requirement to balance the ubiquity of smartphone apps on the one hand with the need to engage in careful phased work that implements appropriate psychological intervention digitally in order to provide meaningful benefit for patients. Results from the current study suggest that participation in the Allay intervention when used in conjunction with continuing pharmacological management of psoriasis, may be of additional benefit for patients.

## CONFLICT OF INTEREST

Prof Fortune has previously undertaken consultancy for AmalgamRx LLC. Dr Clough is Chief Medical Officer at AmalgamRx LLC.

## AUTHOR CONTRIBUTIONS

**D. G. Fortune:** Conceptualization; Data curation; Formal analysis; Funding acquisition; Investigation; Methodology; Project administration; Resources; Supervision; Validation; Visualization; Writing – original draft; Writing – review & editing. **V. Ravnkilde:** Investigation; Project administration; Supervision; Writing – review & editing. **S. Ryan:** Investigation; Project administration; Resources; Supervision; Writing – review & editing. **B. Ramsay:** Investigation; Project administration; Resources; Supervision; Writing – review & editing. **S. Clough:** Conceptualization; Investigation; Project administration; Resources; Supervision; Validation; Visualization; Writing – review & editing. **H. L. Richards:** Conceptualization; data curation; Formal analysis; Investigation; Methodology; Project administration; Visualization; Writing – original draft; Writing – review & editing.

## ETHICS STATEMENT

The study was approved by University of Limerick Hospitals research ethics committee (ref no: 011/018). Written informed consent was obtained from all particpants.

## Data Availability

The data are not publicly available due to privacy or ethical restrictions.

## References

[1] World Health Organization . Global report on psoriasis. Lausanne; 2016. http://apps.who.int/iris/bitstream/10665/204417/1/9789241565189_eng.pdf?ua=1. last accessed 20 August 2021.

[2] Møller AH , Erntoft S , Vinding GR , Jemec GB . A systematic literature review to compare quality of life in psoriasis with other chronic diseases using EQ‐5D‐derived utility values. Patient Relat Outcome Meas. 2015;6:167–77. 10.2147/PROM.S81428 26185476 PMC4500621

[3] Richards HL , Fortune DG , Griffiths CEM , Main CJ . The contribution of perceptions of stigmatisation to disability in patients with psoriasis. J Psychosom Res. 2001;50(1):11–15. 10.1016/S0022-3999(00)00210-5 11259795

[4] Thorneloe RJ , Bundy C , Griffiths CEM , Ashcroft DM , Cordingley L . Adherence to medication in patients with psoriasis: a systematic literature review. Br J Dermatol. 2013;168(1):20–31. 10.1111/bjd.12039 22963128

[5] Fortune DG , Richards HL , Kirby B , McElhone K , Markham T , Rogers S , et al. Psychological distress impairs clearance of psoriasis in patients treated with photochemotherapy. Arch Dermatol. 2003;139(6):752–6. 10.1001/archderm.139.6.752 12810506

[6] All Party Parliamentary Group on Skin . The psychological and social impact of skin diseases on people’s lives; 2013. https://www.appgs.co.uk/publication/view/the‐psychological‐and‐social‐impact‐of‐skindiseases‐on‐peoples‐lives‐final‐report

[7] All Party Parliamentary Group on Skin . Mental health and skin disease; 2020. http://www.appgs.co.uk/publication/view/mental‐health‐and‐skin‐disease

[8] Massoud SH , Alassaf J , Ahmed A , Taylor RE , Bewley A . UK psychodermatology services in 2019: service provision has improved but is still very poor nationally. Clin Exp Dermatol. 2021;46(6):1046–51. 10.1111/ced.14641 33713350

[9] Wheeler M , Guterres S , Bewley AP , Thompson AR . An analysis of qualitative responses from a UK survey of the psychosocial wellbeing of people with skin conditions and their experiences of accessing psychological support. Clin Exp Dermatol. 2022;47(1):37–42. 10.1111/ced.14815 34160837

[10] Xiao Y , Zhang X , Luo D , Kuang Y , Zhu W , Chen X , et al. The efficacy of psychological interventions on psoriasis treatment: a systematic review and meta‐analysis of randomized controlled trials. Psychol Res Behav Manag. 2019;12:97–106. 10.2147/PRBM.S195181 30799963 PMC6369842

[11] Qureshi AA , Awosika O , Baruffi F , Rengifo‐Pardo M , Ehrlich A . Psychological therapies in management of psoriatic skin disease: a systematic review. Am J Clin Dermatol. 2019;20(5):607–24. 10.1007/s40257-019-00437-7 30937679

[12] Zill JM , Christalle E , Tillenburg N , Mrowietz U , Augustin M , Härter M , et al. Effects of psychosocial interventions on patient‐reported outcomes in patients with psoriasis: a systematic review and meta‐analysis. Br J Dermatol. 2018;181(5):939–45. 10.1111/bjd.17272 30291741

[13] Fortune DG , Richards HL , Kirby B , Bowcock S , Main CJ , Griffiths CEM . A cognitive‐behavioural symptom management programme as an adjunct in psoriasis therapy. Br J Dermatol. 2002;146(3):458–65. 10.1046/j.1365-2133.2002.04622.x 11952546

[14] Fortune DG , Richards HL , Griffiths CEM , Main CJ . Targeting cognitive‐behaviour therapy to patients' implicit model of psoriasis: results from a patient preference controlled trial. Br J Clin Psychol. 2004;43(1):65–82. 10.1348/014466504772812977 15005907

[15] Sijercic I , Ennis N , Monson CM . A systematic review of cognitive and behavioral treatments for individuals with psoriasis. J Dermatol Treat. 2020;31(6):631–8. 10.1080/09546634.2019.1690625 31696748

[16] Hudson MP , Thompson AR , Emerson LM . Compassion‐focused self‐help for psychological distress associated with skin conditions: a randomized feasibility trial. Psychol Health. 2020;35(9):1095–114. 10.1080/08870446.2019.1707829 31880167

[17] Maddock A , Hevey D , D’Alton P , Kirby B . A randomized trial of mindfulness‐based cognitive therapy with psoriasis patients. Mindfulness. 2019;10(12):2606–19. 10.1007/s12671-019-01242-3

[18] Fordham B , Griffiths CEM , Bundy C . A pilot study examining mindfulness‐based cognitive therapy in psoriasis. Psychol Health Med. 2015;20(1):121–7. 10.1080/13548506.2014.902483 24684520

[19] Fortune DG , Richards HL , Main CJ , O’Sullivan TM , Griffiths CEM . Developing clinical psychology services in out‐patient dermatology clinics: what factors are associated with non‐uptake of the service? Clin Psychol Forum. 1998;115:34–7.

[20] Bundy C , Pinder B , Bucci S , Reeves D , Griffiths CEM , Tarrier N . A novel, web‐based, psychological intervention for people with psoriasis: the electronic targeted intervention for psoriasis (eTIPs) study. Br J Dermatol. 2013;169(2):329–36. 10.1111/bjd.12350 23551271

[21] van Beugen S , Ferwerda M , Spillekom‐van Koulil S , Smit JV , Zeeuwen‐Franssen ME , Kroft EB , et al. Tailored therapist‐guided internet‐based cognitive behavioral treatment for psoriasis: a randomized controlled trial. Psychother Psychosom. 2016;85(5):297–30. 10.1159/000447267 27508937

[22] Michie S , Yardley L , West R , Patrick K , Greaves F . Developing and evaluating digital interventions to promote behavior change in health and health care: recommendations resulting from an international workshop. J Med Internet Res. 2017;19:e232. 10.2196/jmir.7126 28663162 PMC5509948

[23] Domogalla L , Beck A , Schulze‐Hagen T , Herr R , Benecke J , Schmieder A . Schmieder A Impact of an ehealth smartphone app on the mental health of patients with psoriasis: prospective randomized controlled intervention study. JMIR Mhealth Uhealth. 2021;9(10):e28149. 10.2196/28149 34431478 PMC8576562

[24] Armstrong AW , Ford AR , Chambers CJ , Maverakis E , Dunnick CA , Chren MM , et al. Online care versus in‐person care for improving quality of life in psoriasis: a randomized controlled equivalency trial. J Invest Dermatol. 2019;139(5):1037–44. 10.1016/j.jid.2018.09.039 30481495 PMC6599714

[25] Young PM , Chen AY , Ford AR , Cheng MY , Lane CJ , Armstrong AW . Effects of online care on functional and psychological outcomes in patients with psoriasis: a randomized controlled trial. J Am Acad Dermatol. 2019. 10.1016/j.jaad.2019.05.089 31175908

[26] Finlay AY , Khan GK . Dermatology Life Quality Index (DLQI)‐‐a simple practical measure for routine clinical use. Clin Exp Dermatol. 1994;19:210–16. 10.1111/j.1365-2230.1994.tb01167.x 8033378

[27] Smith BW , Dalen J , Wiggins K , Tooley E , Christopher P , Bernard J . The brief resilience scale: assessing the ability to bounce back. Int J Behav Med. 2008;15(3):194–200. 10.1080/10705500802222972 18696313

[28] Broadbent E , Petrie KJ , Main J , Weinman J . The brief illness perception questionnaire. J Psychosom Res. 2006;60(6):631–7. 10.1016/j.jpsychores.2005.10.020 16731240

[29] Martin M , McCarrier K , Chiou C , Gordon K , Kimball A , Van Voorhees A , et al. Early development and qualitative evidence of content validity for the Psoriasis Symptom Inventory (PSI), a patient‐reported outcome measure of psoriasis symptom severity. J Dermatol Treat. 2013;24(4):255–60. 10.3109/09546634.2012.759639 23249143

[30] Kroenke K , Spitzer RL , Williams JB , Löwe B . An ultra‐brief screening scale for anxiety and depression: the PHQ–4. Psychosomatics. 2009;50(6):613–21. 10.1016/S0033-3182(09)70864-3 19996233

[31] Lamers SM , Westerhof GJ , Bohlmeijer ET , ten Klooster PM , Keyes CL . Evaluating the psychometric properties of the mental health continuum‐short form (MHC‐SF). J Clin Psychol. 2011;67(1):99–110. 10.1002/jclp.20741 20973032

[32] Katie B , Mitchell S . Loving what is: how four questions can change your life. Three Rivers Press; 2003.

[33] Cohen J . Statistical power analysis for the behavioral sciences. Routledge; 2013.

[34] Fortune DG , Richards HL , Kirby B , McElhone K , Main CJ , Griffiths CEM . Successful treatment of psoriasis improves psoriasis‐specific but not more general aspects of patients' well‐being. Br J Dermatol. 2004;151(6):1219–26. 10.1111/j.1365-2133.2004.06222.x 15606518

[35] Maddock A , Hevey D , D’Alton P , Kirby B . Examining individual differences in wellbeing, anxiety and depression in psoriasis using a clinically modified Buddhist psychological model. J Clin Psychol Med Settings. 2020;27:842–58. 10.1007/s10880-019-09686-4 31802330

[36] Meyerowitz‐Katz G , Ravi S , Arnolda L , Feng X , Maberly G , Astell‐Burt T . Rates of attrition and dropout in app‐based interventions for chronic disease: systematic review and meta‐analysis. J Med Internet Res. 2020;22(9):e20283. 10.2196/20283 32990635 PMC7556375

[37] Thompson AR , Guterres S , Bewley AP . Psychodermatological research priorities identified by priority setting partnerships. Clin Exp Dermatol. 2020;45(8):1106–8. 10.1111/ced.14447 32975849

